# Effects of a physical education intervention on cognitive function in young children: randomized controlled pilot study

**DOI:** 10.1186/1471-2431-11-97

**Published:** 2011-10-28

**Authors:** Abigail Fisher, James ME Boyle, James Y Paton, Phillip Tomporowski, Christine Watson, John H McColl, John J Reilly

**Affiliations:** 1 Department of Epidemiology and Public Health, University College London, 1-19, Torrington Place, London, WC1E 6BT, UK; 2 Unit of Lifecourse Nutrition and Health, University of Glasgow, Yorkhill Hospitals, Dalnair Street, Glasgow, G3 8SJ, UK; 3 School of Psychological Sciences & Health, University of Strathclyde, George Street, Glasgow, G1 1QE, UK; 4 Department of Kinesiology, University of Georgia, 115 Ramsey Center, Athens, GA 30602, USA; 5 Education Department, Glasgow City Council, 25 Cochrane Street, Glasgow, G1 1HL, UK; 6 Department of Statistics, University of Glasgow, University Gardens, Glasgow, G12 8QQ, UK

**Keywords:** COGNITION, EXECUTIVE FUNCTION, CHILDREN, PHYSICAL ACTIVITY, EXERCISE

## Abstract

**Background:**

Randomized controlled trials (RCT) are required to test relationships between physical activity and cognition in children, but these must be informed by exploratory studies. This study aimed to inform future RCT by: conducting practical utility and reliability studies to identify appropriate cognitive outcome measures; piloting an RCT of a 10 week physical education (PE) intervention which involved 2 hours per week of aerobically intense PE compared to 2 hours of standard PE (control).

**Methods:**

64 healthy children (mean age 6.2 yrs SD 0.3; 33 boys) recruited from 6 primary schools. Outcome measures were the Cambridge Neuropsychological Test Battery (CANTAB), the Attention Network Test (ANT), the Cognitive Assessment System (CAS) and the short form of the Connor's Parent Rating Scale (CPRS:S). Physical activity was measured habitually and during PE sessions using the Actigraph accelerometer.

**Results:**

Test- retest intraclass correlations from CANTAB Spatial Span (r 0.51) and Spatial Working Memory Errors (0.59) and ANT Reaction Time (0.37) and ANT Accuracy (0.60) were significant, but low. Physical activity was significantly higher during intervention vs. control PE sessions (p < 0.0001). There were no significant differences between intervention and control group changes in CAS scores. Differences between intervention and control groups favoring the intervention were observed for CANTAB Spatial Span, CANTAB Spatial Working Memory Errors, and ANT Accuracy.

**Conclusions:**

The present study has identified practical and age-appropriate cognitive and behavioral outcome measures for future RCT, and identified that schools are willing to increase PE time.

**Trial registration number:**

ISRCTN70853932 (http://www.controlled-trials.com)

## Background

There has been a resurgence of interest in the relationship between physical activity and human cognitive function in recent years [1-5]. Animal evidence suggests that increased physical activity can enhance brain function [6]. Research, largely in older adults, supports the notion that aerobic exercise can enhance human brain structure, prevent age-related brain tissue loss, and improve cognitive performance [7-9]. Aerobic activity may influence executive function specifically [2,7-9].

The literature is consistent in reporting that increased time spent on physical education in schools has no detrimental effect on more 'academic' subjects and may even enhance academic attainment [10-13]. Higher levels of physical fitness in children may be associated with improved neurocognitive processing [12], and increased physical activity may enhance school 'on-task' behavior [13]. Increased physical activity may therefore provide cognitive and educational benefits across childhood and adolescence. Experimental evidence in children is very limited [3,5]: < 1% of published exercise and cognition studies have involved child participants, and experimental evidence from pre-school children is absent. There is therefore a need for randomized trials in children to establish definitively the presence of any cognitive effects of physical activity and to identify their nature (e.g. dose-response effects; specificity to particular cognitive processes). The UK Medical Research Council framework for complex interventions suggests that prior to carrying out full RCT it is important to carry out exploratory trials to examine: acceptability of study measurements; feasibility of the proposed trial; likely retention of participants and missing data [14].

The measurement of executive function is especially problematic in younger children [15]. Testing for effects of exercise on executive function in children is also problematic because of the difficulty of establishing exercise programs that effectively increase the levels of physical activity [16-18]. Carrying out well designed and adequately powered RCT to test for relationships between physical activity and executive function in young children therefore presents researchers with a number of major challenges. Future RCT in this area will have to be informed by exploratory studies. These studies need to establish which interventions are practical and what is the optimal 'dose' of physical activity. They will also need to establish the practical utility and reliability of potential measures of executive function (outcome measures) in young children. Finally, they will need to provide information on effect sizes to calculate sample size and ensure adequate power of future large scale RCT.

The present study therefore aimed to collect the data necessary to design and power a future school-based RCT on the influence of aerobic activity on executive function in 5-6 year olds. All cognitive measures were specified *a priori *as candidate measures potentially sensitive to changes in physical activity.

## Methods

### Participants and Methods

Healthy children, attending the second year of 6 mainstream primary schools were invited to take part in the reliability study, the practical utility study, and the exploratory RCT. Parents gave informed written consent to participation in the study and children provided verbal assent and an initialled consent form. The study was approved by the UK Central Office for Research Ethics (COREC). Participants were recruited from entire year 2 classes of the 6 volunteering schools in the City of Glasgow, Scotland. Children were eligible for inclusion in the study (n 185 eligible) if they had no known diagnosed disorder of cognition, and had no physical condition affecting their ability to participate in a school PE program (assessed by parent questionnaire).

### Study Design

The present study was in two phases: an initial study of practical utility and reliability of the cognitive outcome measures over three weeks, followed by a 10 week exploratory RCT.

### Psychological Measures

A literature search and contact with experts in the field prior to the present study suggested three measures of cognition which might be suitable as candidate outcome measures for an RCT in young children: the Cognitive Assessment System (CAS;[19]); the Cambridge Neuropsychological Test Battery (CANTAB http://www.cantab.com); the Attention Network Test (ANT) [20]. High reliability of measurements in children under age 7 has been reported in one study for the CAS [19], but such data are not available for the ANT or the CANTAB and so reliability data for the ANT and CANTAB were collected in the present study prior to the exploratory RCT.

All cognitive tests were administered to children individually in a quiet room in school using a laptop and touch-screen. E-Prime Software (http://www.psnet.com) was used with the ANT. All children were tested by the same trained researcher (AF), seated comfortably approximately 53 cm from the laptop screen, and with the dominant hand resting on the computer mouse. For a detailed description of the ANT see http://www.sacklerinstitute.org/users/jin.fan and Rueda et al [20], but in brief, the ANT was administered in four blocks of tests, each lasting approximately 5 minutes: a practice block of tests was used first to train the children in what was expected, and to identify any problems they had in performing the test (e.g. understanding of what to do/how to do it); in three subsequent blocks which formed the basis of the ANT outcomes in the present study children were asked to perform 48 short tests, each of which involved a 'flanker' (a fish) presented in 12 potential states (congruent, incongruent, neutral; with no cue, a central cue, a double cue, or a down/up cue). After appearance of the fish on the laptop screen children were asked to press the right or left mouse button corresponding to the direction the fish was pointing. The outcomes for the ANT were reaction time to the stimulus of the fish on screen (ms) and accuracy (number of times the correct mouse button was selected).

For a detailed description of the CANTAB see http://www.cantab.com. For the present study the CANTAB working memory battery was administered as recommended by the manufacturer (http://www.cantab.com), incorporating a test of Spatial Memory Span (SSP) and a test of Spatial Working Memory (SWM). A motor screening test was carried out prior to CANTAB administration to ensure no visual or comprehension problems, and to familiarise participants with the study procedures. The motor screening test involved the appearance of a pink cross on a black laptop screen and children were asked to touch the center of the cross with the dominant hand. The SWM tests the number of items which can be held in working memory by asking participants to observe the laptop screen as a pattern of boxes appears, then to remember and replicate the pattern by touching boxes which are displayed on the screen. The SWM starts with two boxes (items) and progresses to a maximum of 9 boxes, but after two failed attempts the SWM ends. The SSP involves presentation of colored squares on the laptop screen and tests the ability of participants to remember the longest sequences of squares which appear ('span length' test), the ' total errors' (the number of times an incorrect box is chosen) and 'total usage errors' (the number of times boxes are chosen out of the sequence in which they were presented).

The CAS is better established for use in children and adults than the other two cognitive tests [19]. In the present study it was administered precisely as recommended by the test authors [19]. It has to be administered by Psychologists and involves four sub-scales, each tested using three assessments on the laptop: planning; attention; perceptual processing; memory.

In order to collect test-retest reliability data and to test for changes associated with the intervention in the exploratory RCT, the ANT and CANTAB data were collected on three occasions: 3 weeks before to the intervention; just before the intervention (week 0, just before the intervention began) and following the 10 week intervention or control conditions (end of week 10). Since encouraging reliability data were available for children of this age with the CAS [19], and resources were limited, it was decided not to collect reliability data for the CAS and administer it only at weeks 0 and 10 for the exploratory RCT. Research psychologists responsible for administering the CAS, and research assistants entering the pre and post ANT and CANTAB data, were blinded to group allocation, and to the nature of the study.

### Intervention Study: Intervention and Control Group Allocation and Treatment

Immediately after collection of retest data at week 0, a statistician independent of the present study randomised the six schools by computer to receive either the Intervention or Control PE for ten weeks. Prior to randomisation the schools had been matched pair-wise to provide three pairs of schools with similar socio-economic profile, assessed using an area based measure; [21], size, geographical location, and availability of space for PE. The local council PE specialists responsible for all public primary schools in Glasgow were asked to devise a 10 week experimental PE curriculum for the intervention which consisted solely of the most aerobically active components of the existing curriculum. The same PE specialists delivered 1 session per week and the usual classroom teacher delivered the other session in the experimental group. Teachers received training in the experimental Intervention PE programme and were encouraged to make the sessions 'as physically active as possible' 'minimise instruction time', and 'minimise/avoid any time children were waiting to use equipment, or standing around; minimise object control tasks'.

There is evidence that numerous psychological variables can change as a result of any intervention, perhaps related to increased attention being paid to study participants. To control for any improvement in psychological variables by simply intervening [22] and to try to ensure that any differences between groups might be attributable to the difference in aerobically intense PE between the two groups, control and intervention groups were matched for intervention time. To match conditions in the intervention group over the same 10 week period the three schools randomly allocated to the control condition received the standard Scottish elementary school PE curriculum, but PE was increased from 1 to two hours per week for the 10 week study, and 1 of the 2 hours of PE per week was delivered by a specialist and one by the class teacher in both groups. To reduce risk of bias of parent ratings of behavior and participant expectations children and parents were not informed which group was hypothesised to change, and outcome measures were made blind to group allocation. During the 10 week winter school term in which the present study took place standard PE consisted largely of skill development (e.g. object control - throwing and catching a ball, balance). The lack of emphasis on aerobic activities increased the contrast between intervention and control groups. Physical activity was measured by accelerometry during the sessions. The PE sessions were observed directly by researchers in two randomly selected teacher-directed and two specialist-directed intervention classes in the first two weeks of the intervention. The direct observations were made to identify problems in the implementation of the intervention, to answer questions about the intervention, and to encourage delivery of the PE intervention as 'prescribed'.

### Objectively measured physical activity and sedentary behavior

Habitual physical activity data were collected at week 0 (baseline) by asking participating children to wear the Actigraph GT1M accelerometer (http://www.theactigraph.com) for 7 days. Actigraphs were worn over the right hip on a waist belt and used as described previously [23-25] with 1 minute epochs. Evidence based cut points [23-25] were applied to accelerometry output to define sedentary behavior (accelerometer counts per minute < 1100; [23], light intensity physical activity (accelerometer count per minute 1100-3200) and moderate-vigorous intensity physical activity (MVPA, > 3200 counts per minute) [25]. Use of cut-points and epochs varies widely between studies, but the options selected for the present study were age-appropriate and choice of cut-point and epoch has only a small impact on the measurement of time spent sedentary and time spent in MVPA [24]. Validity of the Actigraph in children has been demonstrated repeatedly against criterion methods of energy expenditure and direct observation [24,26]. Reliability of Actigraph-measured habitual physical activity in Scottish 5-6 year olds is high so long as at least three days of data are collected [27] and in the present study accelerometry data were excluded if < 3 days and 9 hours each day were obtained. Only data collected between the hours of 7 am and 11 pm were included in analyses.

### Statistical analysis and power

#### Reliability of the ANT and CANTAB

Intraclass correlations (ICC), and standard error of measurement (SEM) - measures of within subject variation from biological difference or equipment 'noise' or error [28] were calculated. The coefficient of variation (CV) - the standard deviation expressed as a percentage of the mean, and limits of agreement (LOA) were calculated using SPSS software and the reliability spreadsheet http://www.sportsci.org/resource/stats. While the required level of reliability of a test will depend on the application, there is general agreement in the psychological literature that ICC's should exceed 0.75 [29].

#### Exploratory RCT

The exploratory RCT examined changes in the cognitive outcomes measured by the ANT, CANTAB, and CAS. We also measured parent ratings of child behavior using the short form of the Conner's Parent Rating Scale [30], on the grounds that increases in physical activity could have favorable effects [31]. All data were checked for normal distribution using graphical summary of data, assessment of skewness, descriptive statistics, and tests of normality. For initial between-group comparisons of cognitive data, t tests were carried out on the change in variables over time. A general linear model was applied to all psychological and behavioral outcome measures with the follow up score as the response variable, 'group' (Intervention or Control), socio-economic status (SES), gender, school (nested within group) as factors and age and baseline (week 0) score as covariates. The study was a pilot, intended to produce data necessary to adequately power a full scale RCT, so was not powered formally. However, a sample of around 60 children (30 per group) was considered both practical and adequate for an exploratory study.

## Results

### Characteristics of study participants and flow through the trial

#### Reliability and practical utility studies

A total of 71 children and their parents consented to participation and were eligible. Of these, three were absent from school on the days scheduled for testing with the ANT and CANTAB, providing initial data on 68 children for the reliability and practical utility studies. Of these 68, a further 4 children were absent from school on days scheduled for the retest, giving a final sample of 64 children for test-retest data for the CANTAB (29 girls, 35 boys; mean age 6.2 years, SD 0.3). A further 2 children were excluded from the ANT analysis as their reaction times were < 200 ms indicating anticipatory responding (pressing the mouse button before the onset of stimulus), giving a sample of 62 participants for the reliability study for the ANT. Characteristics of participants are given in Table 1.

**Table 1:** Baseline characteristics of study participants (n 64)

	Intervention	Control	Total
Variable	Group	Group	Sample
Age, years	6.1 (0.3)	6.2 (0.3)	6.1 (0.3)
Body mass index z-score	0.30 (1.01)	0.48 (1.30)	0.38 (1.16)
Boys (%)	47%	42%	45%
Left-handed (%)	12%	8%	9%
Birth-weight (kg)	3.2 (0.6)	3.1 (0.5)	3.1 (0.6)
SES category	6 (1)	7 (1)	7 (1)

1. No significant differences between groups at baseline (just before intervention started).

2. BMI z-scores calculated relative to UK 1990 reference data.

3. Birth-weight data obtained by maternal report.

4. SES socioeconomic status -categorical variable based on postcode from highest SES (category 1) to lowest (category 7) [21]

#### Exploratory RCT

**Figure 1:**
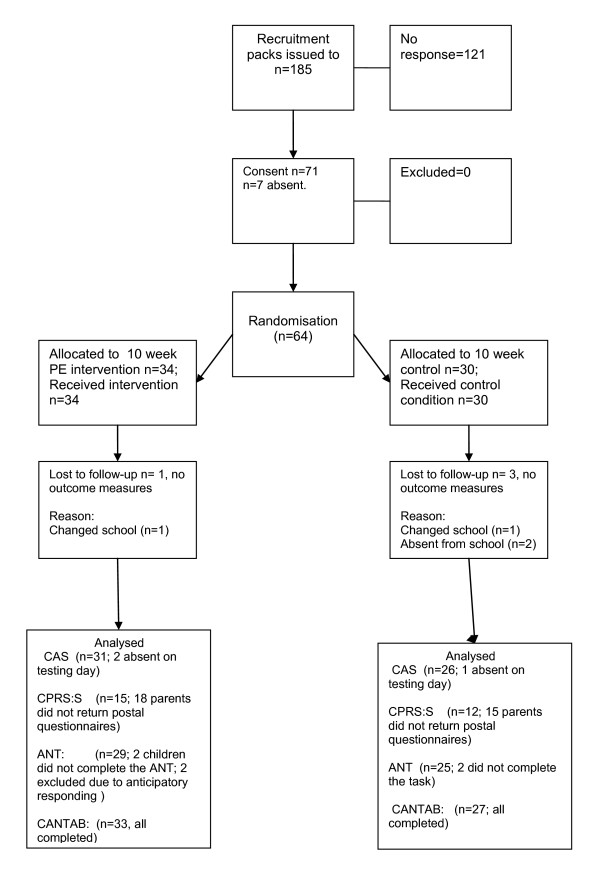
**Study flow diagram for the exploratory randomised controlled trial**.

Flow of participants through the trial is described in Figure 1. Baseline (week 0) differences in characteristics between intervention and control groups were not significant. Habitual and PE class physical activity data are shown in Table 2.

**Table 2:** Objectively measured habitual physical activity and sedentary behavior at baseline, and during control and intervention PE classes (median, IQR)

	Intervention group	Control group	Total sample
Total volume of habitual physical activity at baseline, accelerometry cpm	721 (632,799)	654 (580,809)	691 (602,799)
Total volume of physical activity (cpm) during PE sessions	1801 (1618, 2173)*	1158 (1057, 1501)	N/A
Baseline % of habitual time in MVPA	3 (2,5)	3 (2, 5)	3 (2,5)
% of PE session in MVPA	20 (14, 27)*	9 (7,15)	N/A
Baseline % of habitual time in sedentary behavior	78 (73, 80)	80 (75, 82)	78 (74, 81)
% PE session in sedentary behavior	44 (36, 49)*	61 (52, 65)	N/A

1.MVPA; moderate-vigorous intensity physical activity

2.* Significantly different from control group at P < 0.001.

#### Reliability and practical utility study results

Test-retest reliability data for the CANTAB and ANT are provided in Table 3. For the CANTAB and ANT none of the proposed outcomes reached the a priori criterion of acceptability [29]. Children's compliance to the CANTAB and ANT was generally very good. The time taken to complete the testing ranged from 30-39 minutes for the entire ANT, and for the CANTAB varied by scale (2-3 minutes for Motor Screening Test; 3-6 minutes for Spatial Span; 10-15 minutes for Spatial Working Memory).

**Table 3:** Test Retest Reliability of the CANTAB and ANT, mean (SD)

	Measure 1 3 weeks prior to exploratory RCT baseline	Measure 2 In the week the RCT started	ICC	SEM	CV	LOA (+/-)
**CANTAB **(n = 64)						
Spatial span length	3.51 (0.86)	3.35 (0.84)	0.51*	0.60	19.65	1.70
Spatial span total errors	10.36 (4.28)	9.10 (3.97)	-0.08	4.30	14.84	12.15
Spatial span usage errors	2.87 (1.78)	2.66 (2.26)	0.11	1.20	51.13	5.43
Spatial working memory errors	68.93 (11.04)	69.53 (10.37)	0.59*	7.19	7.65	20.36
Spatial working memory strategy	39.35 (2.54)	38.63 (2.72)	0.03	2.67	7.78	7.57
**ANT **(n = 62)						
ANT reaction time (ms)	960.33 (138.19)	855.84 (122.93)	0.37*	104.23	16.77	297.27
ANT accuracy	103.91 (27.95)	105.81 (28.86)	0.60**	18.21	83.60	51.96

Cambridge Neuropsychological Test Battery (CANTAB) Spatial span length: (number of items that can be held in short term memory), Spatial span total errors (the number of times an incorrect item is selected), Spatial span usage errors; the number of times an item is selected out of sequence. Spatial working memory strategy: whether a strategic search is used. Attention Network Test (ANT) Reaction Time: to stimulus in milliseconds (mean of median), stimulus is a fish appearing on screen (mouse button is pressed); Accuracy: total correct from 144; a 'correct' response is when the mouse button pressed corresponds with the direction the 'flanker' (fish) is pointing; ICC - intraclass correlation; SEM - standard error of measurement; CV - coefficient of variation; LOA - Limits of agreement. *P < 0.05 **P < 0.001.

#### Exploratory RCT results

Compliance of schools with the intervention and control PE was good - in all six schools all 20 of the 'prescribed' sessions were implemented (based on a diary kept by class teachers) during the 10 week study. Total physical activity was significantly greater during the intervention than control sessions (median difference 649 counts per minute; P < 0.001; Table 2). Time spent in MVPA was significantly higher in intervention than in control sessions (P < 0.001), however this equated to approximately only 12 minutes MVPA per 1 hour PE lesson in the intervention group (at 20% of time spent in MVPA) versus 5 minutes MVPA per 1 hour PE lesson in the control sessions (at 9% of time spent in MVPA; Table 2). The percentage of time spent sedentary during PE was significantly lower in intervention than control PE sessions (P < 0.001). However, overall percentage time spent sedentary was high in both the intervention and control sessions (44% and 61% respectively).

Scores and unadjusted and adjusted cognitive and behavioral outcome analyses from week 0 to 10 week are summarised in Table 4. There were no significant between group differences in any of the CAS scales (all p > 0.05). The CANTAB Spatial Working Memory Error rate was significantly reduced in the Intervention group in both unadjusted analysis and adjusted analysis. In the unadjusted analysis, scores on subscales of the CPRS: the Cognitive Problems/Inattention, Hyperactivity and ADHD index were significantly lower post Intervention than Control. In the adjusted model only the between-group differences for the Cognitive Problems and Inattention scale remained significant.

**Table 4:** Exploratory RCT: Cognitive and Behavioral Outcomes (mean, SD)

	Intervention Group		Control Group		P-values for Between Group Difference	
Outcome	Baseline	Follow-up	Baseline	Follow-up	Unadjusted	Adjusted*
**Cognitive Assessment Scale (CAS)**						
Planning	106 (17)	113 (17)	118 (11)	125 (10)	NS	NS
Attention	103 (13)	106 (10)	108 (15)	110 (14)	NS	NS
Simultaneous	99 (13)	104 (12)	106 (17)	106 (15)	NS	NS
Successive	103 (12)	108 (16)	102 (16)	105 (21)	NS	NS
Full Scale	104 (14)	110 (9)	113 (15)	116 (13)	NS	NS
**Attention Network Test (ANT)**						
Reaction time (mean, ms)	982 (169)	864 (123)	923 (113)	804 (43)	NS	NS
Accuracy (correct from 144)	96 (24)	110 (23)	113 (4)	113 (13)	0.01^a^	0.06
**CANTAB**						
Spatial span	3 (1)	3 (1)	4 (1)	3 (1)	0.002^b^	0.02
Spatial working memory errors	72 (11)	64 (14)	66 (14)	67 (10)	0.009^c^	0.01
**Conner's Behavioral Rating Scale**	n = 15		n = 12			
Oppositional	53 (7)	46 (4)	51 (9)	52 (12)	0.05^d^	NS
Cognitive problems/Inattention	54 (7)	48 (4)	51 (5)	52 (7)	0.02^e^	0.01
Hyperactivity	57 (13)	52 (6)	55 (9)	57 (13)	0.04^f^	NS
ADHD Index	53 (6)	49 (5)	50 (3)	51 (5)	0.04^g^	NS

*Adjusted analyses from general linear models with follow up (week 10 value) 'group' (intervention or control), socio-economic status (SES), gender and school as factors and age and baseline score as covariates.

a. Mean difference 14, 95%CI 3,29.

b. Mean difference 1, 95% CI 0,1.

c. Mean difference -7, 95% CI -3,-15.

d. Mean difference -8, 95% CI 0, -10.

e. Mean difference -6, 95% CI -1, -8.

f. Mean difference -6, 95% CI -1, -9.

d. Mean difference -5, 95% CI 0,-7.

## Discussion

### Main findings and study implications

If causal links between physical activity, including PE, and cognition are established in children and adolescents then educators and policy makers may be more receptive to the promotion of physical activity. Establishing such links will require evidence from experimental studies, and obtaining more definitive evidence of this kind will require exploratory trials upon which more definitive trials are based [14].

The present study suggests that the cognitive and behavioral measures used were practical in this sample and setting. Each cognitive test battery was completed in less than 40 minutes and compliance with study procedures was generally high. Reduced compliance with the Conner's Parents Rating Scale may have resulted from resource limitations of the present study (inability to send out second mailings to parents), or may indicate that this scale has low practical utility and may be unsuitable for future studies. Reliability of the cognitive measures was more of a concern. Reliability of the CANTAB and ANT scales, with intraclass correlations between 0.37 and 0.60, were lower than would generally be considered acceptable (29), and this may limit their usefulness as outcome measures in future RCT. While empirical data from young children are scarce, effect sizes may be too small to be detectable with measures of the reliability observed in the present study [3,5,7]. The present study provides information sufficient to power future trials for a range of cognitive outcomes. For example, the mean difference between groups in the CAS 'Planning' subscale was 7 with an SD of 15. At 80% power and p = 0.05, a sample of 74 per group would be adequate to detect effects of a magnitude which could not be explained by test-retest differences in the CAS measure.

The intention in the present study was to develop a PE intervention that delivered around 40 minutes of MVPA within every hour-long PE session, and in this respect the pilot study was not successful. Future RCT should reduce the amount of time children spend not moving during PE, and increase the amount of MVPA. In the study by Davis et al [2] a high intensity of physical activity was achieved by a well chosen activity program, the use of real time heart-rate data to provide feedback on the intensity of physical activity, and the presence of a number of research assistants to instruct each class. These characteristics of the study by Davis et al [2] ensured high fidelity to the prescribed dose of physical activity, but would present challenges to translation of the intervention to the school or after-school setting. The ability to develop and implement an enhanced PE intervention is likely to be critical to any future RCT in this area. Previous studies have noted repeatedly that levels of physical activity during PE are often very low, and have also noted the difficulty in producing sustained increases in intensity of physical activity during PE in children [11,16-18,32]. An essential part of process evaluation of future RCT will be to examine whether or not the 'prescribed' levels of physical activity are actually being reached by children: accelerometry was adequate for this purpose but did not provide teachers or children with real time feedback on the intensity of PE.

### Comparisons with other evidence

The present studies are not directly comparable with other literature, though the RCT of an after-school based physical activity intervention in sedentary overweight and obese older children and adolescents by Davis et al [2] is the most readily comparable study. When designing the present study, outcome data from Davis et al. were not available, and even if they had been available their generalisability to children who were on average more than three years younger was unclear. There was no guidance on how intense, and how frequent an intervention for a younger age group should be in terms of enhancing cognitive function, or what would be acceptable to primary schools in a different population.

### Study Limitations

While sample sizes in the studies described here were relatively small, they were adequate to examine reliability and practical utility of the outcome measures chosen, were adequate for an exploratory RCT, and sufficient to power more definitive RCT in future. The number of tests carried out will have increased the probability of significant differences being observed by chance. It should also be noted that some of the significant differences which favored the intervention group in the present study were observed with small sample sizes and with outcome measures which had low reliability (e.g. ANT accuracy) -the results of the current study should therefore be viewed with caution.

In the present study we lacked the resources to assess the reliability of the CAS, which required administration by psychologists, and at the time of the study we depended on a single study to support the view that reliability of the CAS was high. Naglieri and Das [19] measured reliability of the CAS in a nationally representative sample of 872 US children and adolescents, age 5-17 years, and reliabilities of all CAS subscales in all age groups ranged from 0.83-0.93 [19]. An assessment of the reliability of the CAS in our sample and setting would have been helpful, and reasons why the reliability of CANTAB and ANT were lower in the present study than reliability previously reported for the CAS are unclear.

While time spent in MVPA was significantly higher in the intervention than in the control group PE classes, both spent a large proportion of time sedentary. Several biologically plausible mechanisms link physical activity and cognition [4,6,33] but identifying these in future larger-scale and longer-term RCT may require a greater contrast in the 'dose' of physical activity between experimental and control groups. Additional research on the nature of existing PE-before the present study intervention-would have been helpful in designing a PE program which was more physically active. A further difficulty arises from lack of certainty in the optimal accelerometry cut-points. With lower cut-points than those used in the present study levels of apparent MVPA would have been higher, and levels of apparent sedentary behavior lower, in both the intervention and control groups [24].

The present study was designed with longer-term translation to school systems in mind (the rationale for choosing PE as the means of delivering the physical activity intervention). However, any longer-term translation of this sort of intervention to schools would need to be informed by evidence which the present study did not address, such as needs assessments and qualitative studies with teachers and school pupils.

## Conclusions

The cognitive and behavioral effects of increases in physical activity in children merit greater emphasis in research because of the enormous potential for short and long term academic and health benefits [33]. The greater degree of neural plasticity of young children means that they may have most to gain from increased physical activity, but studying physical activity-cognition relationships in young children is especially challenging. The present studies have provided evidence that should inform the future RCT which will be necessary in order to better understand relationships between physical activity and cognition in young children in future.

## Conflict of interests

The authors declare that they have no competing interests.

## Authors' contributions

All authors were responsible for study concept & design, and all were involved in study data analysis & interpretation. AF and CW collected data. All authors were involved in drafting & revision of the manuscript for important intellectual content. All authors were responsible for final approval of manuscript submission.

## Pre-publication history

The pre-publication history for this paper can be accessed here:

http://www.biomedcentral.com/1471-2431/11/97/prepub
